# Age‐Related Declines in Cerebral Blood Flow are Moderated by Sex, but not APOE ε4, in Mild Cognitive Impairment

**DOI:** 10.1002/alz.092731

**Published:** 2025-01-09

**Authors:** Danielle L Sanchez, Maria G Sabbah, Ilana J Bennett

**Affiliations:** ^1^ University of California, Riverside, Riverside, CA USA

## Abstract

**Background:**

Age‐related disruptions in cerebral blood flow have been linked to increased Alzheimer’s disease risk. At least one study in cognitively unimpaired (CU) older adults found that cerebral blood flow varies with interactive effects of age, sex, and Apolipoprotein (APOE) ε4 status. However, it remains unknown whether these effects are stronger in older adults diagnosed with mild cognitive impairment (MCI). Therefore, the current study examined the moderating effects of sex and APOE ε4 status on age‐related differences in cerebral blood flow separately in CU and MCI groups.

**Method:**

Perfusion‐ and T1‐weighted magnetic resonance imaging data and APOE ε4 status (ε4+, ε4‐) were obtained from 111 CU and 49 MCI Alzheimer’s Disease Neuroimaging Initiative (ADNI3) participants at their baseline visit. Cerebral blood flow was measured as the average perfusion within anatomical masks of bilateral cortical lobes (frontal, temporal, parietal, occipital) and the hippocampus. Multiple linear regressions tested the effect of each moderator (sex, APOE ε4 status) on the relationship between age and cerebral blood flow separately in each region of interest and each cognitive status group.

**Result:**

For the MCI group, results revealed both independent and interactive effects of Age and Sex on cerebral blood flow in the frontal, parietal, and occipital lobes, with the age‐related decline in cerebral blood flow being stronger in males than females. There were no significant effects of Age, Sex, or their interaction on cerebral blood flow in the hippocampus. Moreover, there were no significant effects of APOE ε4 status on cerebral blood flow nor moderation of Age on cerebral blood flow in any region. For the CU group, there were no significant effects of Age, Sex, APOE ε4 status, or their interactions on cerebral blood flow.

**Conclusion:**

The age‐related decline in cortical lobe cerebral blood flow was stronger in males with clinically diagnosed MCI. This finding indicates that sex and cognitive status, but not APOE ε4 status, are important moderators of cerebrovascular health.

